# Physical Mapping of *Pm57*, a Powdery Mildew Resistance Gene Derived from *Aegilops searsii*

**DOI:** 10.3390/ijms21010322

**Published:** 2020-01-03

**Authors:** Zhenjie Dong, Xiubin Tian, Chao Ma, Qing Xia, Beilin Wang, Qifan Chen, Sunish K. Sehgal, Bernd Friebe, Huanhuan Li, Wenxuan Liu

**Affiliations:** 1 National Key Laboratory of Wheat and Maize Crop Science, College of Life Sciences, Henan Agricultural University, Zhengzhou 450002, China; zhenjiedong@hotmail.com (Z.D.); XiubinTian2019@hotmail.com (X.T.); machao0813@hotmail.com (C.M.); qingxia412@hotmail.com (Q.X.); Anlieen0109@hotmail.com (B.W.); chenqifan02@163.com (Q.C.); 2 Department of Agronomy, Horticulture and Plant Science, South Dakota State University, Brookings, SD 57007, USA; sunish.sehgal@sdstate.edu; 3 Wheat Genetic and Genomic Resources Center, Department of Plant Pathology, Throckmorton Plant Sciences Center, Kansas State University, Manhattan, KS 66506-5502, USA; friebe@ksu.edu

**Keywords:** *Pm57*, physical mapping, RNA-seq, common wheat, molecular markers

## Abstract

Powdery mildew caused by *Blumeria graminis* f. sp. *tritici* (*Bgt*) is one of many severe diseases that threaten bread wheat (*Triticum aestivum* L.) yield and quality worldwide. The discovery and deployment of powdery mildew resistance genes (*Pm*) can prevent this disease epidemic in wheat. In a previous study, we transferred the powdery mildew resistance gene *Pm57* from *Aegilops searsii* into common wheat and cytogenetically mapped the gene in a chromosome region with the fraction length (FL) 0.75–0.87, which represents 12% segment of the long arm of chromosome 2S^s^#1. In this study, we performed RNA-seq using RNA extracted from leaf samples of three infected and mock-infected wheat-*Ae*. *searsii* 2S^s^#1 introgression lines at 0, 12, 24, and 48 h after inoculation with *Bgt* isolates. Then we designed 79 molecular markers based on transcriptome sequences and physically mapped them to *Ae*. *searsii* chromosome 2S^s^#1- in seven intervals. We used these markers to identify 46 wheat-*Ae*. *searsii* 2S^s^#1 recombinants induced by *ph1b*, a deletion mutant of pairing homologous (*Ph*) genes. After analyzing the 46 *ph1b*-induced 2S^s^#1L recombinants in the region where *Pm57* is located with different *Bgt*-responses, we physically mapped *Pm57* gene on the long arm of 2S^s^#1 in a 5.13 Mb genomic region, which was flanked by markers *X67593* (773.72 Mb) and *X62492* (778.85 Mb). By comparative synteny analysis of the corresponding region on chromosome 2B in Chinese Spring (*T.* *aestivum* L.) with other model species, we identified ten genes that are putative plant defense-related (R) genes which includes six coiled-coil nucleotide-binding site-leucine-rich repeat (CNL), three nucleotide-binding site-leucine-rich repeat (NL) and a leucine-rich receptor-like repeat (RLP) encoding proteins. This study will lay a foundation for cloning of *Pm57*, and benefit the understanding of interactions between resistance genes of wheat and powdery mildew pathogens.

## 1. Introduction

Common wheat (*Triticum aestivum* L., 2n = 6x = 42, AABBDD) is the most widely grown food crop, a cash crop with world trade greater than that of all other crops combined, and also a worldwide staple food, providing valuable plant protein for humans. Wheat is susceptible to more than 30 diseases, of which powdery mildew, caused by the fungus *Blumeria graminis* f. sp. *tritici* (*Bgt*), can lead to severe yield losses and grain quality degradation in a relatively short period of time [1,2,3]. Development and deployment of wheat varieties with resistance to powdery mildew is considered the most effective, economical, and environmentally friendly way to control losses caused by this disease [4,5]. Currently, up to 89 resistance genes/alleles, *Pm1* to *Pm65*, are cataloged [6,7,8,9,10,11]. Some of these resistance genes, such as *Pm8* derived from *Secale* *cereale* L. (2n = 2x = 14, RR), and *Pm21*, from *Dasypyrum villosum* (L.) P. Candargy (2n = 2x = 14, VV), have been used in wheat breeding programs worldwide [12,13,14,15]. However, up to now, only a few powdery mildew resistance (*Pm*) genes have been used successfully in wheat cultivars because of deleterious traits associated with these genes, especially those derived from wild relatives of wheat [9]. Further with the emergence of novel viral *Bgt* isolates have led to a continuous breakdown of *Pm* gene resistance widely deployed in wheat cultivars. Thus, the discovery, identification, and deployment of new and effective *Pm* genes are vital for successful wheat production.

Isolation of plant resistance genes (R-genes) benefits the understanding of the molecular mechanisms involved in R-gene-mediated disease resistance and accelerates the discovery of more R-genes. Since the first R-gene *Hm1* was cloned from *Zea mays* in 1992, the number of cloned R-genes has increased steadily [16,17]. This knowledge and germplasm will help us to design resistance crop varieties for the future [18,19,20,21,22]. Currently, map-based positional cloning, in which the genomic location and R-gene sequence are identified through closely linked molecular markers, is recognized as a conventional and efficient way by which many R-genes were isolated successfully. One crucial step in map-based cloning procedures is to generate high density physical and genetic maps in the target gene region [21,23]. This method is usually expensive and time-consuming, particularly for the genes in partially/less sequenced or complex polyploid genomes such as wheat [24]. Mapping alien genes from wild relatives of wheat is a challenge due to the rare homoeologous recombination between alien chromosomes from wild species and their homoeologous counterparts in wheat, which was strictly controlled by pairing homoeologous (*Ph*) genes in hexaploid wheat, limited genome sequence references, and alien genome-specific molecular markers [25,26,27]. Availability of increased reference sequences from common wheat (AABBDD) and its progenitor species *T.* *turgidum* ssp. *dicoccoides* (Körn. ex Asch. & Graebner) Thell. (2n = 4x = 28, AABB), *T. urartu* Tumanian ex Gandilyan (2n = 2x = 14, AA), and *Ae. tauschii* Coss. (2n = 2x = 14, DD), together with advances in RNA sequencing (RNA-seq) and bulked segregant RNA-seq (BSR-seq) have greatly enhanced the pace of conventional map-based cloning of R-genes from wheat relatives [28,29,30,31,32,33,34,35]. For example, up till now at least five cataloged *Pm* genes including *Pm2*, *Pm3b*, *Pm8*, *Pm21*, *Pm60* have been cloned. Gene *Pm3b* from hexaploid wheat, *Pm21* from *D*. *villosum* and *Pm60* from *T. urartu* were isolated using map-based cloning strategy or integrated with other gene cloning approaches such as cloning through isolation and sequencing of flow-sorted mutant chromosome (MutChromSeq), or rapid cloning through R-gene enrichment and sequencing (RenSeq) which captures the nucleotide-binding leucine-rich repeats (NLR) R-genes [36,37,38,39,40,41].

Transcriptome sequencing or RNA-seq can provide extensive data pre and post-pathogen infection for the analysis and has been widely used to discover R-genes and reveal the transcriptional expression pattern of genes involved in pathogen-defense responsive pathways in crops including hexaploid wheat [28,32,42,43,44,45]. For example, *Stpk-V*, a putative serine/threonine-protein kinase gene derived from *D. villosum* against powdery mildew was successfully cloned and characterized based on integrating microarray analysis of transcriptomes before and after *Bgt*-inoculation with physical mapping of chromosomal segment locating *Pm21* [44]. Further RNA-seq has also been used to develop molecular markers for diagnosing chromosomes, chromosome segments or genes in wild relatives [46,47].

*Pm57*, a gene conferring resistance to powdery mildew, was derived from *Ae. searsii* Feldman & Kislev ex Hammer (2n = 2x = 14, S^s^S^s^), one of the S-genome diploid species belonging to the section *Sitopsis* (Jaub. & Spach) Zhuk. [48]. We previously transferred this gene into common wheat based on *ph1b*-induced homoeologous recombination between wheat and *Ae*. *searsii*, and located the gene at the long arm of chromosome 2S^s^#1 at an interval of fraction length (FL) 0.75–0.87 by comparison of the chromosome recombination breakpoints of the resistant and susceptible recombinants [7]. In the current study, we have performed RNA-seq using Chinese Spring (CS)-*Ae*. *searsii* introgression lines to develop 2S^s^#1 specific markers and identified *ph1b*-induced 2S^s^#1 recombinants to physically map *Pm57* into a small genomic region.

## 2. Results

### 2.1. RNA-seq Quantity, Sequence Assembly, and Differential Expression Gene (DEGs) Analysis

A total of 94.88–111.83 × 10^6^ high-quality reads, which constituted 10.19–16.35 Gb of the cDNA sequences were generated for each sample in this study. Sequencing quality scores, Q30, which infers a base call accuracy of 99.90%, were more than 93.49% for each sample, signifying that RNA-seq quality in the study is sufficient for subsequent sequence assembly. The high-quality reads were further assembled into 71,313 unigenes (46.87 Gb) of 331 bp median length, ranging from 201 to 63,334 bp in length, using the short-read assembly software Trinity [49].

Pairwise comparison of the unigenes in CS at 0 h post-inoculation (hpi) using *Bgt* isolates (RNA-seq library referred as A-CS) and at 12–48 hpi (RNA-seq library referred as M-CS) to those in line 2011-400 carrying *Pm57* (A-400 for a RNA-seq library at 0 hpi, and M-400 for a library at 12–48 hpi). Based on the threshold of reads per kilobase per million mapped reads (RPKM) value of (A-400 or M-400) > 0.1 & (A-CS and M-CS) = 0, a total of 6,444 (9.04%) DEGs were selected as candidate unigenes specific to the CS-*Ae*. *searsii* disomic 2S^s^#1 addition line (2011-400 with accession ID TA3581) which were uniquely presented in 2011-400, but absent in CS (Supplementary Table S1). The 3,587 (5.03%) DEGs shared by the three CS-*Ae*. *searsii* 2S^s^#1 introgression line (2011-400, 89-88, and 89-69) with a threshold of RPKM value of (A-400 or M-400) > 0.1 & (A-88 or M-88) > 0.1 & (A-69 or M-69) > 0.1 were selected as candidates specific to 2S^s^#1 segment of FL0.67–0.87 on the long arm of chromosome 2S^s^#1 where the *Pm57* gene is mapped (Supplementary Table S2). Comparing the unigenes of all three *Pm57* lines (2011-400, 89-88, and 89-69) against those of CS (Supplementary Tables S1 and S2), 500 candidate DEGs were selected for the design of specific PCR primers for chromosome 2S^s^#1 and 2S^s^#1 long-arm segment of FL0.67–0.87.

### 2.2. Validation of DEGs by Quantitative RT-PCR (qRT-PCR)

**Figure 1:**
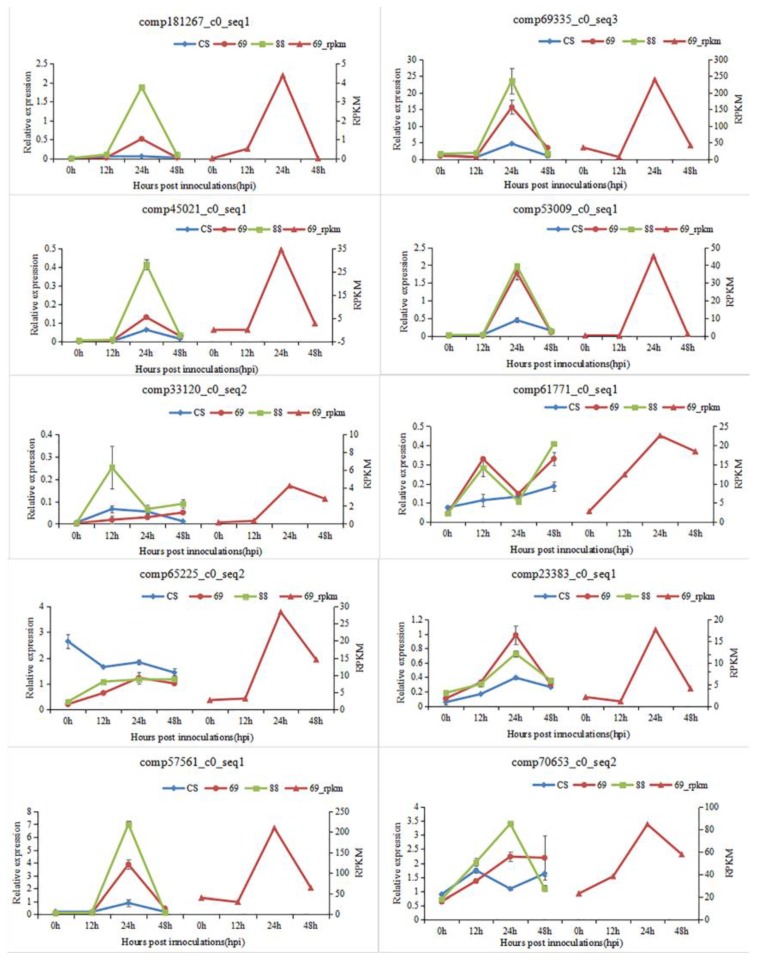
Validation of RNA sequencing results by qRT-PCR. Gene expression displaying on the left side of each graph resulted from qRT-PCR using specific primer pairs listed in Supplementary Table S3; gene expression of line 89-69 (in red color) on the right side of each graph was based on analysis of RNA sequencing data in each photo.

To validate the DEGs based on RNA-seq analysis, 10 *Bgt*-infection induced DEGs described as “plant disease resistance-related protein” in the protein family (Pfam) database were further evaluated by qRT-PCR in the *Bgt*-resistant recombinants 89-88 and 89-69, and susceptible CS. Primer pairs were designed for qRT-PCR for each of the 10 DEGs (Supplementary Table S3). The qRT-PCR results (Figure 1, left side of each graph) showed that expression levels of seven (comp181267_c0, comp69335_c0, comp45021_c0, comp53009_c0, comp23383_c0, comp57561_c0 and comp70653_c0) of ten DEGs reached to a maximum at 24 hpi. One gene (comp33120_c0) was highest at 12 hpi. The unigene comp61771_c0 caused significant up-regulation at 12 hpi and 48 hpi, whereas comp65225_c0 was up-regulated at three time points (12, 24 and 48 hpi) in resistant lines, but down-regulated in the susceptible CS (Figure 1, Supplementary Table S4). Although gene expression levels of line 89-69 evaluated by qRT-PCR were not exactly the same as those identified based on RNA-seq analyses, gene comp61771_co of line 89-69, which showed highest expression levels at 24 and 48 hpi based on qRT-PCR, was identified to show maximum expression level at 24 hpi by RNA-seq analysis, but expression patterns of the remaining nine genes were consistent with those of line 89-69 (Figure 1, right side of each graph). These results of RNA-seq and qRT-PCR analyses confirmed the robustness and reproducibility of this study.

### 2.3. Chromosome Fraction Length Interval Assignment of Chromosome 2S^s^#1-Specific Markers

**Figure 2:**
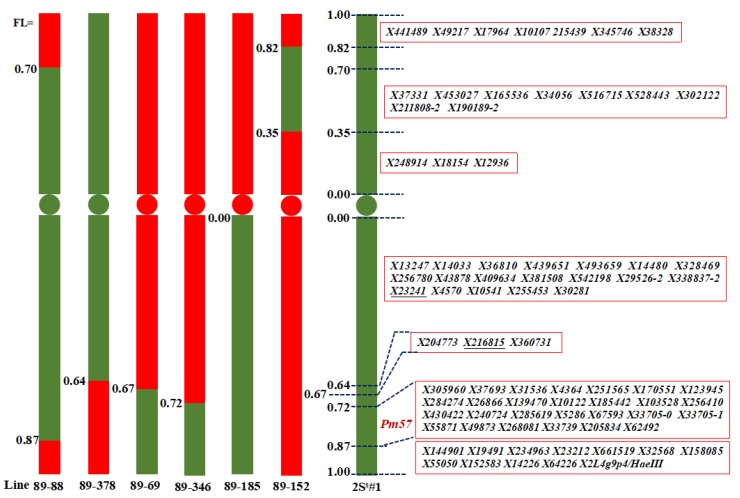
Physical mapping of *Ae*. *searsii* chromosome 2S^s^#1 specific molecular markers to different fraction length intervals. Numbers on the left of each chromosome represent the fraction length (FL) of recombination. Lines 89-88, 89-69, 89–346, and 89–185 carried *Pm57*, which mapped the gene at LFL0.72–0.87; Marker names located at each interval are in the red boxes on the right of chromosome 2S^s^#1; Chromatin of *Ae*. *searsii* is displayed in green color and wheat in red color. No specific markers were found at SFL0.70–0.82 and LFL0.67–0.72 of 2S^s^#1. Markers *X23241* and *X216815* underlined are used for subsequent selection of putative recombinants in the F_2_ segregating population.

**Figure 3:**
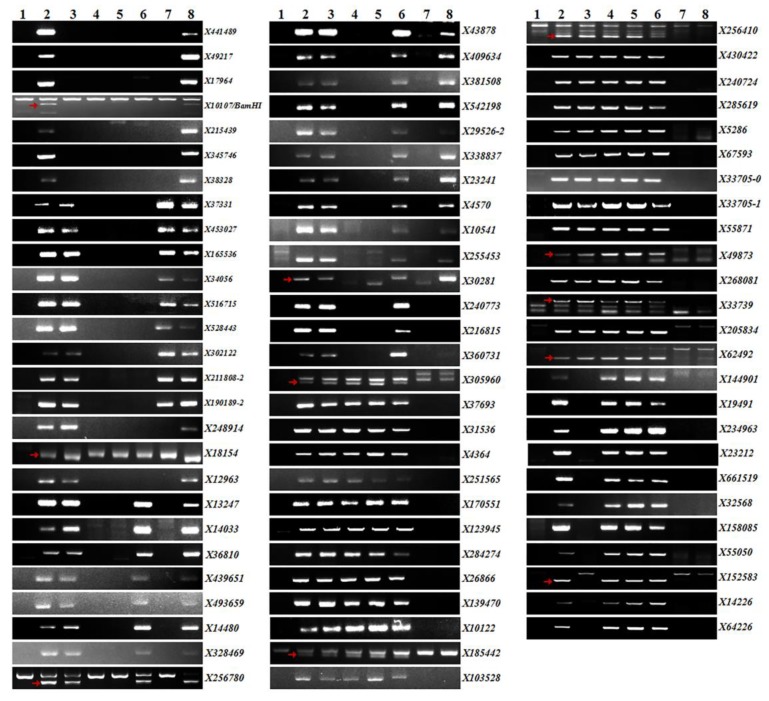
Electrophoresis patterns of 79 2S^s^#1-specific molecular markers identified in this study. Lanes: 1, CS; 2, CS-*Ae*. *searsii* 2S^s^#1 disomic addition line (2011-400); 3-6, CS-*Ae*. *searsii* 2S^s^#1 recombinant 89-88, 89-69, 89–346, and 89–185, which carried *Pm57*, respectively; 7–8, CS-*Ae*. *searsii* 2S^s^#1 recombinants 89–152 and 89–378 with no *Pm57* genes. The arrows indicate PCR amplification bands of 2S^s^#1 specificity while more than a band amplified.

Chromosome 2S^s^#1 was divided into 9 FLs by comparing recombination breakpoints of CS, disomic 2S^s^#1 addition line 2011-400, and six other 2S^s^#1 recombinants (Table 1), which includes four intervals of short-arm fraction length (SFL) SFL0.82–1.00, SFL0.70–0.82, SFL0.35–0.70 and SFLC-0.35 and five intervals of the long-arm fraction length (LFL) LFLC-0.64, LFL0.64–0.67, LFL0.67–0.72, LFL0.72–0.87 and LFL0.87–1.00 (Figure 2). PCR analysis of 500 primers pairs on the recombinants led to the assignment of 79 molecular markers into seven of the nine FLs (Supplementary Table S5). 27 molecular markers were located at LFL0.72–0.87, where *Pm57* was mapped, of which 3 makers (*X139470*, *X10122* and *X33705–1*) were unstable, thus not used in subsequent physical mapping of *Pm57*. The other markers were mapped to SFL0.82–1.00 (7), SFL0.35–0.70 (9), SFLC-0.35 (3), LFLC-0.64 (19), LFL0.64–0.67 (3), and LFL0.87–1.00 (11) on chromosome 2S^s^#1. No specific markers could be mapped to two intervals, SFL0.70–0.82 and LFL0.67–0.72, of chromosome 2S^s^#1 in this study (Figure 2 and Figure 3).

### 2.4. Development of CS-Ae. searsii Disomic 2S^s^#1 Recombinants and Physical Mapping of Pm57

To physically map *Pm57* we screened 2280 individuals of an F_2_ population of TA3809/recombinant 89-88 segregating for *Pm57* in a homozygous *ph1b* background using only two molecular markers, *X23241* and *X216815*, located at LFLC-0.64 and LFL0.64–0.67, respectively, in the long arm of 2S^s^#1 (Supplementary Table S6), and *Bgt*-responsive assay of the individuals. A total of 46 CS-*Ae*. *searsii* 2S^s^#1 recombinants were identified based on the presence of either or both markers *X23241* and *X216815* (for *Bgt*-susceptible individuals)*,* and absence of the markers (for *Bgt*-resistant plants). These recombinants, which included 28 resistant and 18 susceptible recombinant lines, formed a secondary recombinant population for subsequent physical mapping of *Pm57*, along with lines 89-88, 2011-400, and CS as checks. All recombinants were genotyped using 28 chromosome 2S^s^#1-specific molecular markers, of which 24 markers were located at LFL0.72–0.87 where *Pm57* is mapped, and 4 at *Pm57* flanking intervals of LFLC-0.64 (1), LFL0.64–0.67 (1), and LFL0.87–1.00 (2), respectively (Table 2, Supplementary Tables S5 and S6). Comparison of molecular marker order at 2S^s^#1 interval of LFL0.72–0.87 with the corresponding genomic block of chromosome 2B of *T*. *aestivum* revealed collinearity except for markers *X26866* and *X251565* at current loci 7 and 10, respectively.

**Figure 4:**
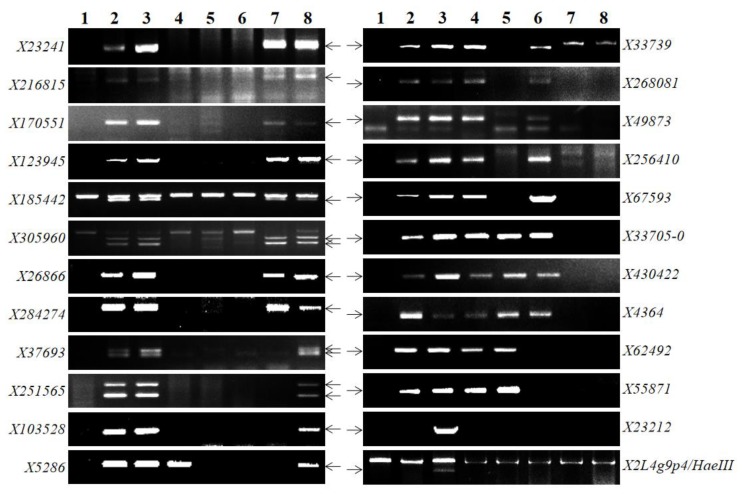
Electrophoresis patterns of 24 molecular markers at 2S^s^#1 interval locating *Pm57*. Lane 1, *Bgt*-susceptible control CS; 2-3, *Bgt*-resistant controls 89-88, and 2011-400; 4-6, *Bgt*-resistant recombinants of CS-*Ae*. *searsii* 2S^s^#1, 88R-1-12-2, 88R-3-19-1, and 88R-5-4-2; 7-8, *Bgt*-susceptible recombinants 88S-18-3-2, and 88S-17-4-1. Arrows indicate 2S^s^# 1 specific amplicons.

**Figure 5:**
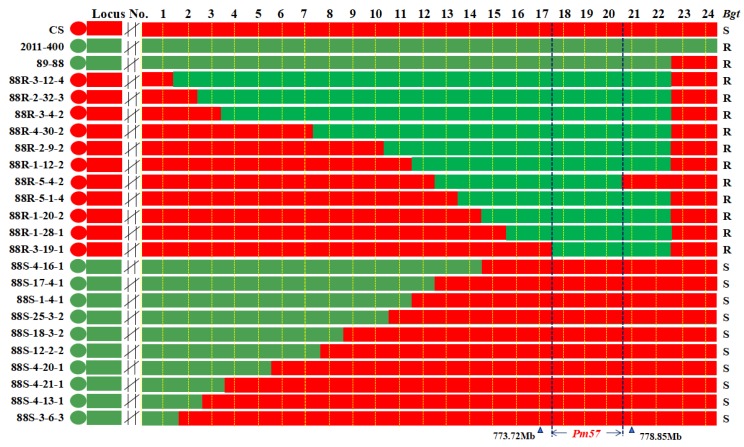
Molecular marker and *Bgt*-response among different CS-*Ae*. *searsii* 2S^s^#1 recombinant lines. Markers at loci 1-24 are listed in Table 2. Genomic positions are corresponding to chromosome 2B of CS RefSeq v1.1 (IWGSC, 2018). Molecular markers (1-24) sequences, derived unigenes, and fraction length intervals are in Supplementary Table S5. Only 21 recombinant types which include 11 resistant and 10 susceptible recombinants from 46 newly identified CS-*Ae*. *searsii* recombinants are displayed (Supplementary Table S6). The chromatin of *Ae*. *searsii* is in green color and wheat in red. The genomic region, flanked by markers *X67593* at 773.72 Mb at locus 17 and *X62492* at 778.85 Mb at locus 21, is shared by all the resistant recombinants, but absent in all susceptible recombinants, thus delimiting *Pm57* to the 5.13 Mb genomic region.

Analyses of these 28 markers and the *Bgt*-responses of the recombinants classified the 46 CS-*Ae. searsii* recombinants into 21 types with different recombination breakpoints, including 11 types as resistant and 10 types of susceptible recombinants (Figure 4 and Figure 5). Molecular marker analysis showed that resistant recombinant 88R-3-19-1 had the shortest 2S^s^#1 genomic region < 9.88 Mb locating *Pm57*, between markers *X67593* at locus 17 (773.72 Mb) and *X23212* at locus 23 (783.60 Mb). *Bgt*-resistant recombinant 88R-5-4-2 was the only one missing the 2S^s^#1 segment harboring distal markers *X6249* at locus 21 (778.85 Mb) and *X55871* at locus 22 (779.34 Mb) (Figure 4). The unique genomic regions shared by all 28 *Bgt*-resistant recombinants, and absent from all 18 susceptible recombinants, are the genomic regions with markers *X33705*, *X430422/X285619/X205834/X240724*, and *X4364* at loci 18, 19, and 20, respectively. Thus, *Pm57* was physically mapped to a maximum genomic region of 5.13 Mb flanked by markers *X67593* (773.72 Mb) at locus 17 and *X62492* (778.85 Mb) at locus 21, respectively (Figure 5).

### 2.5. Comparative Synteny and Genes in Pm57 Candidate Region

**Figure 6:**
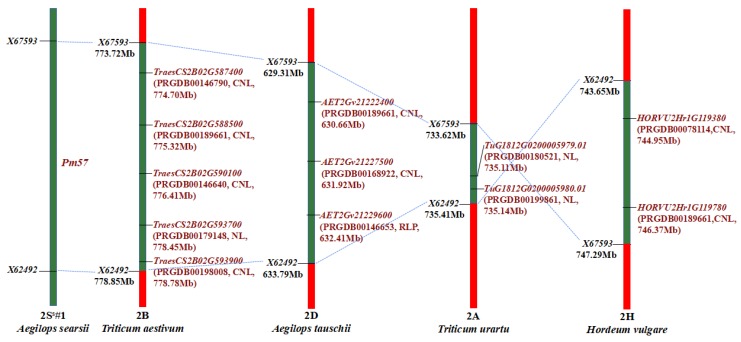
Comparative analysis of *Pm57* syntenic region and R-genes in *T. aestivum*, *Ae. tauschii*, *T. urartu*, *H. vulgare*. Syntenic blocks on each chromosome are displayed in green. Markers flanking synteny regions are on the left; names, classes, and genomic positions of the R-genes are on the right of each chromosome. Chromosome inversion was observed in this region-based markers *X67593* to *X62492* at chromosome 2H of *H. vulgare* when compared with all other species.

For the discovery of putative resistance gene (R-gene) candidates of *Pm57*, we conducted a synteny comparison with related species using the markers *X67593* at 773.72 Mb and *X62492* at 778.85 Mb flanking the 5.13 Mb *Pm57* genomic region. The 5.13 Mb syntenic region corresponded to genomic regions of 5.13 Mb, 4.48 Mb, 3.64 Mb, 1.79 Mb, and 212.61 kb on chromosomes 2B, 2D, 2H, 2A, and Bd5 from *T*. *aestivum* (72 genes), *Ae. tauschii* (70 genes), *Hordeum vulgare* (123 genes), *T. urartu* (42 genes), and *Brachypodium distachyon* (38 genes), respectively. Though a total of 345 genes were identified in the syntenic blocks in different species where *Pm57* mapped only 12 of these genes were annotated as putative R-genes in the PRGdb database (http://prgdb.crg.eu/wiki/Main_Page). Of these 12 R-genes, 8 genes, 4 from *T. aestivum* (*TraesCS2B02G587400*, *TraesCS2B02G588500*, *TraesCS2B02G590100* and *TraesCS2B02G593900*), 2 from *Ae. tauschii* (*AET2Gv21222400* and *AET2Gv21227500*), and 2 from *H*. *vulgare* (*HORVU2Hr1G119780* and *HORVU2Hr1G119380*), are CNL class R-genes encoding proteins with at least a coiled-coil domain, a nucleotide-binding site and a leucine-rich repeat (CC-NB-LRR). The other 2 genes *TuG1812G0200005979.01*, and *TuG1812G0200005980.01* from *T*. *urartu*, and one gene *TraesCS2B02G593700* from *T. aestivum* are NL class R-genes encoding proteins consist of nucleotide-binding subdomain at N-terminal and leucine-rich repeat at the C-terminal, but lack of coiled-coil structures. Additionally, one gene *AET2Gv21229600* from *Ae. tauschii* annotated as RLP class R-gene encoding protein contains leucine-rich receptor-like repeat, a transmembrane region of 25AA, and a short cytoplasmic region was also identified in the syntenic region. None of the genes from *B*. *distachyon* were annotated as putative R-genes even though up to 38 genes were located in the syntenic blocks (Figure 6, Supplementary Table S7). Of the 8 CNL class R-genes, 3 genes, gene *TraesCS2B02G588500* from *T. aestivum*, *AET2Gv21222400* from *Ae. tauschii,* and *HORVU2Hr1G119780* from *H*. *vulgare* are all annotated to a putative R-gene PRGDB00189661 (*Hv.31127*) in class CNL, decreasing the number of CNL class R-genes from 8 to 6 different genes (Figure 6). Moreover, chromosome inversion was identified between markers *X67593* and *X62492* at chromosome 2H of *Hordeum vulgare* based on synteny comparison with group 2 chromosomes 2A, 2B, 2D of *T*. *aestivum*, 2A of *T*. *urartu*, and 2D of *Ae. tauschii.*

## 3. Discussion

Powdery mildew causes significant losses to wheat production worldwide. Exploring the gene pools of wheat wild relatives for new and durable *Bgt*-resistance could lead to the development of wheat cultivars with robust powdery mildew resistance. However, mapping, characterization, and finally cloning R-genes derived from hexaploid wheat, which has three closely related and independent genomes (A, B and D), is challenging due a huge genome size estimated at ~17 Gb [32,42,43]. This effort is further complicated if the genes are originating from wheat wild relatives as a result of low wheat-alien homoeologous recombination that is strictly controlled by *Ph* genes in hexaploid wheat genetic backgrounds. The other limitation is the availability of alien genome-specific molecular markers due to the lack of reference genomic sequences in the wild wheat species.

To deal with these problems of alien gene mapping, we first performed RNA sequencing using three wheat-*Ae*. *searsii* chromosome 2S^s^#1 introgression lines (TA3581, 89-88, and 89-69) carrying *Pm57* and a susceptible control CS, which provided abundant transcriptome sequences for the development of *Ae*. *searsii* chromosome 2S^s^#1 specific molecular markers. Here we physically mapped 27 markers specific to chromosome 2S^s^#1L at the fraction length interval of LFL0.72-0.87 where *Pm57* gene is mapped, of which 24 markers were consistent and used for subsequent physical map of *Pm57*, together with other 4 markers at flanking intervals. Secondly, we constructed a mapping population with a genetic background of homozygous *ph1b* genes that enhance the homoeologous recombination of chromosome 2S^s^#1 with its homoeologous counterpart in wheat. This resulted in the identification of 46 CS-*Ae*. *searsii* recombinants, which provide useful materials for subsequent physical mapping of *Pm57*.

By analysis of 46 *ph1b*-induced 2S^s^#1L recombinants with different *Bgt*-responses using 24 2S^s^#1L molecular markers at the LFL interval where *Pm57* is located, and 4 markers at flanking intervals of LFLC-0.64, LFL0.64-0.67, and LFL0.87-1.00, we physically mapped *Pm57* to the long arm of 2S^s^#1 in a 5.13-Mb genomic region flanked by markers *X67593* at 773.72 Mb and *X62492* at 778.85 Mb. In this 5.13-Mb genomic region, a total of 12 putative plant defense-related genes (R-genes) were discovered based on comparative synteny analysis of *T*. *aestivum*, *T*. *urartu*, *Ae*. *tauschii*, *H*. *vulgare*, and *B*. *distachyon*.

Of the 90 cataloged *Pm* genes, at least five, including *Pm2*, *Pm3b*, *Pm8*, *Pm21*, and *Pm60*, have been successfully isolated using different gene cloning approaches. Map-based cloning was used for *Pm3b*, *Pm60*, and *Pm21*; homologous cloning for *Pm8*, which is homologous locus to *Pm3b*; an R-gene mutant-located chromosome flow sorting and sequencing (MutChromSeq) for *Pm2,* and resistance gene enrichment sequencing (RenSeq) and Pacific Biosciences single-molecule real-time sequencing for *Pm21* isolation [8,15,36,37,39,41]. Although isolated by different strategies, all the *Pm* genes currently cloned are coiled-coil, nucleotide-binding site, leucine-rich repeat (CNL) class R-genes, which initiate effector-triggered immunity usually accompanied by local cell death, also known as the hypersensitive response [52,53,54]. In this study, a total of 12 putative R-gene candidates were discovered locating in collinear genomic regions, of which 8 R-genes, including 4 genes from *T. aestivum* (*TraesCS2B02G587400*, *TraesCS2B02G588500*, *TraesCS2B02G590100* and *TraesCS2B02G593900),* 2 from *Ae. tauschii* (*AET2Gv21222400* and *AET2Gv21227500*), and another two from *H*. *vulgare* (*HORVU2Hr1G119780* and *HORVU2Hr1G119380*), are CNL type R-genes, to which all cloned *Pm* genes belong. Furthermore, three of these CNL type R-genes (*TraesCS2B02G588500*, *AET2Gv21222400* and *HORVU2Hr1G119780*) are likely homologs (PRGDB00189661, *Hv.31127*). Thus, these six CNL type R-genes in the syntenic region could be potential candidates for *Pm57* for further cloning of *Bgt*-resistance gene derived from *Ae*. *searsii*. Functional validation of *Bgt*-resistance of these six CNL type R-genes using virus-induced gene silencing (VIGS) technology is under progress.

## 4. Materials and Methods

### 4.1. Plant Materials

A total of 9 lines were used in this study, including the wheat landrace CS (TA3808), a CS *ph1b* mutant stock (TA3809) lacking the *Ph1* gene and thereby permitting homoeologous recombination, a CS-*Ae*. *searsii* disomic 2S^s^#1 addition line (2011-400, accession ID TA3581) where a pair of chromosome 2S^s^#1 were added into CS genetic background (CS-DA 2S^s^#1), and 6 CS-*Ae*. *searsii* disomic 2S^s^#1 recombinant stocks [7] (Table 1). The #1 designation is used to distinguish between the same *Ae*. *searsii* chromosome derived from different *Ae*. *searsii* accessions [55]. All materials, except the CS-*Ae*. *searsii* disomic 2S^s^#1 recombinant stocks are kindly provided by the Wheat Genetics Resource Center (WGRC) at Kansas State University, USA and maintained at the Experimental Station of Henan Agricultural University, China.

Of the materials listed in Table 1, three wheat-*Ae*. *searsii* chromosome 2S^s^#1 introgression lines, TA3581, 89-88 and TA5109 (i.e., 89-69) carrying *Pm57* highly resistant to powdery mildew and the susceptible control CS, were used for RNA-seq in this study. The recombinant 89-88 had a pair of recombined chromosomes where the distal parts of the short and long arms of 2S^s^#1 were replaced by homoeologous segments derived from wheat chromosome 2A (Ti2AS-2S^s^#1S·2S^s^#1L-2AL). TA5109 (i.e., 89-69) was a recombinant stock with the distal 33% of the 2S^s^#1L segment replacing homoeologous parts of wheat 2BL (T2BS·2BL-2S^s^#1L) [7].

For chromosome interval mapping of 2S^s^#1-specific markers, a total of 8 lines, which includes CS, TA3581, 89-88, TA5109, and other 4 wheat-*Ae*. *searsii* chromosome 2S^s^#1 recombinants with different sizes of 2S^s^#1L segments (89–346, 89–185, 89–152 and 89–378), were used to assign molecular markers to different fraction length intervals at chromosome 2S^s^#1.

### 4.2. Construction of cDNA Libraries for Illumina Sequencing

Three wheat-*Ae*. *searsii* chromosome 2S^s^#1 introgression lines with *Bgt*-resistance, including TA3581, 89-88 and TA5109 (i.e., 89-69) carrying *Pm57* and the susceptible control CS, were used for RNA-seq in this study. *Pm57* was mapped at the interval of LFL0.72–0.87 on 2S^s^#1, which was shared by these three introgression lines based on comparison of the recombination breakpoints of the lines.

Seedling growing, fresh leaf collection for RNA sample preparation for cDNA library construction follow the procedures described by Li et al. (2019) [56]. Total RNA was extracted from leaves of three wheat-*Ae*. *searsii* chromosome 2S^s^#1 introgression lines (2011-400, 89-88, and 89-69) carrying *Pm57* and the wheat landrace CS at 0, 12, 24, and 48 hpi independently using Trizol reagent (Cat. No. B511311, Sangon Biotech (Shanghai) Co., Ltd.), generating 16 RNA samples, 4 samples at different *Bgt*-infection time points for each of 4 genotypes. RNA quality and purity were inspected using a Nanodrop spectrophotometer (Thermo Fisher Scientific Inc., Wilmington, USA) and 1% formaldehyde gel electrophoresis. After quality assessments, equal amounts of qualified RNAs at 12, 24, and 48 hpi were combined for each line, thus, producing 4 pools of RNAs referred as M-XX, including M-CS for CS, M-400 for 2S^s^#1 addition line 2011-400, M-88, and M-69 for recombinants 89-88 and 89-69, respectively, and 4 RNA samples at 0 hpi referred as A-XX, which includes A-CS, A-400, A-88, and A-69. Moreover, the RNA of line 89-69 at time points 0, 12, 24 and 48 hpi (referred as A-69, C-69, D-69, and F-69, respectively) were used separately for subsequent construction of sequencing cDNA libraries and RNA-seq analysis at various *Bgt*-infection time points.

Construction of 12 RNA-seq libraries, which includes 4 derived from RNA at 0 hpi (A-CS, A-400, A-88, and A-69), 4 from a mixture of equal amounts of RNA at 12, 24 and 48 hpi (M-CS, M-400, M-88, and M-69) of CS, 2011-400, 89-88, and 89-69, respectively, together with 4 RNA samples from the recombinant 89-69 at 0, 12, 24 and 48 hpi (A-69, C-69, D-69, and F-69), and Illumina RNA sequencing were performed by LC-Bio (Hangzhou), China. The generated paired-end reads were used for downstream sequence assembly using Trinity software [49], and transcriptome data analyses after removal of sequences containing adapters or poly-N above 5%, reads less than 100 bp in length and those of low quality.

### 4.3. RNA-seq Data Analysis

The assembled sequences were designed as unigenes. Read counts of unigenes were normalized as RPKM. All the unigenes were assigned to chromosomes or chromosome arms based on blastn alignment against wheat reference genomic sequences (Wheat_IWGSC_RefSeq_v1_chromosomes) with a cutoff of expect ≤ 1e-10 and qcov ≧ 75% at URGI BLAST (https://urgi.versailles.inra.fr/blast/blast.php).

Annotation and gene function categories of the unigenes were assigned with a cut off e-value < 10^−10^ based on Blastp alignment against protein sequences in 6 public databases including NR (NCBI non-reduction protein sequences, https://blast.ncbi.nlm.nih.gov/Blast.cgi?PROGRAM=blastn&PAGE_TYPE=BlastSearch&LINK_LOC=blasthome), Pfam (Protein family, http://pfam.sanger.ac.uk/), UniProtKB/Swiss-Prot (a high-quality manually annotated non-redundant protein sequence database, http://www.uniprot.org/statistics/Swiss-Prot), KEGG (Kyoto encyclopedia of genes and genomes, http://www.kegg.jp/), COG (Clusters of orthologous groups of proteins) and GO (Gene Ontology, http://www.geneontology.org/), and PRGdb (Plant resistance gene database) (http://prgdb.org; http://prgdb.crg.eu/wiki) [57].

### 4.4. Validations of RNA-seq Data by Quantitative RT-PCR

cDNA from *Bgt*-inoculated seedling leaves of CS, 89-88, and 89-69 at time points 0, 12, 24 and 48 hpi were used to validate RNA-seq data by quantitative RT-PCR (qRT-PCR). Total RNA was isolated using Trizol reagent (Cat. No. B511311, Sangon Biotech (Shanghai) Co., Ltd., China). A total of 1–2 µg RNA of each sample was used to synthesize first-strand cDNA using a Thermo Scientific RevertAid First-Strand cDNA Synthesis Kit (Cat. no. K1622, Thermo Fisher Scientific Inc., USA) following the manufacturer’s instructions. The reverse transcription product was checked by PCR using GAPDH primer sets.

10 DEGs, shared by all introgression lines carrying *Pm57* in the RNA-seq analysis, were randomly selected to validate gene expression patterns by SYBR Green real-time RT-PCR using QuantiNova SYBR Green PCR Kit (Cat. no. 208054, Qiagen, USA). The qRT-PCR reaction mixture contained 200 ng cDNA templates, 0.67 μM primer sets, and 1× QuantiNova SYBR Green PCR Master Mix. qRT-PCR amplification was performed on Bio-Rad iQ5 Real-Time PCR System (Bio-Rad Laboratories, Inc., USA) under conditions as follows: 95 °C, 5 min, 40 cycles at 95 °C, 10 s, 60 °C, 30 s for cycle threshold (Ct) value calculation. A melting curve was performed 1 cycle at 95 °C, 15 s, 60 °C, 1 min, 71 cycles at 60 °C, 15 s. Amplified products were finally maintained at 16 °C, 10 min. Each reaction was repeated three times with three biological replicates. The *actin* gene of wheat was used as the endogenous control and the expression level changes of each gene were calculated according to the method of Livak et al. (2001) [58]. Sequences of the primer sets of the selected and *actin* gene were designed using the software Primer Premier 5 (PREMIER Biosoft, CA, USA) and were listed in Supplementary Table S3.

### 4.5. Molecular Marker Analysis

Genomic DNA (gDNA) was isolated from 5–10 cm segments of young leaves with a DNeasy Plant Mini Kit (Qiagen, Cat No. 69104) following the instruction guide. PCR primer pairs specific for 2S^s^#1 were designed based on RNA-seq sequences of 2S^s^#1 which uniquely presented in 2011-400 but in CS, and sequences shared by three 2S^s^#1 introgression lines 2011-400, 89-88, and 89-69. The PCR reaction mixture preparation and PCR amplification by “Touch-down 63” followed Li et al. (2019) and Liu et al. (2017) [7,59], respectively. PCR products were resolved in 1.5% agarose gels and visualized by ethidium bromide staining by a Tanon 2500 Gel Imaging System (Tanon Science & Technology Co., Ltd., Shanghai, China).

### 4.6. Powdery Mildew Response Assay

Powdery mildew assays were conducted by using a mixture of *Bgt* isolates (composite) collected in the Henan Province and were kindly provided by Yuli Song in Henan Academy of Agricultural Sciences. The *Bgt* composite was inoculated after the first leaf of each seedling had fully unfolded and maintained in a controlled greenhouse with a daily cycle of 14 h light at 22 ± 2 °C and 10 h of darkness at 18 ± 2 °C. Powdery mildew infection types (IT) were scored 7–10 days post-inoculation, when the susceptible controls were heavily infected. A 0–4 IT scale was used, where 0 = no visible symptoms; 0; = hypersensitive necrotic flecks; 1 = small and sparse conidial development; 2 = colonies with moderately developed hyphae, but few conidia; 3 = colonies with well-developed hyphae and abundant conidia, but colonies not joined; and 4 = colonies with well-developed hyphae and abundant conidia with mostly overlapping colonies. Plants with ITs 0–2 were considered resistant, whereas those with ITs 3–4 were susceptible [5,60].

### 4.7. Identification of CS-Ae. searsii 2S^s^#1 Recombinants and Physical Mapping of Pm57

The CS-*ph1b* mutant stock TA3809 was crossed with the 89-88 recombinant, which had a pair of recombined chromosomes where the distal parts of the short and long arms of 2S^s^#1 were replaced by homoeologous segments derived from wheat chromosome 2A (Ti2AS-2S^s^#1S·2S^s^#1L-2AL), to generate F_1_ hybrids that contained a monosomic recombined chromosome Ti2AS-2S^s^#1S·2S^s^#1L-2AL in a homozygous *ph1b* background. The F_2_ segregating population derived from self-pollinated F_1_ hybrids were used for selection of 2S^s^#1 recombinants using 2S^s^#1-specific molecular markers *X23241* (488.87 Mb) and *X216815* (658.61 Mb)*,* which located at 2S^s^#1 intervals of LFLC-0.64 and LFL0.67–0.72 neighboring to the centromere, respectively.

After completing *Bgt*-responsive assays of the recombinants, *Bgt*-resistant F_2_ individuals lacking either or both of the markers *X23241* and *X216815*, should contain the new interstitially recombined chromosome (Ti2AS.2AL-2S^s^#1L-2AL), and the *Bgt*-susceptible, presenting either or both of the markers, were selected to compose a secondary mapping population for further mapping of *Pm57* using more markers at the interval of LFL0.72–0.87, where *Pm57* mapped based on the RNA-seq sequences.

## Figures and Tables

**Table 1:** List of plant materials used in this study.

WGRC ^1^ Accession Number	Description	Fraction Length Interval Harboring 2Ss#1 Segment	Reference
TA3808	CS	- ^2^	- ^3^
TA3809	CS *ph1b* mutant	- ^2^	Sears (1977) [50]
TA3581 (2011-400)	CS-*Ae. searsii* disomic 2S^s^#1 addition line	- ^2^	Friebe et al. (1995) [51]
TA5109 (89-69)	CS-*Ae. searsii* T2BS.2BL-2S^s^#1L recombinant line	LFL0.67-1.00	Liu et al. (2017) [7]
89-88	CS-*Ae. searsii* Ti2AS-2S^s^#1S.2S^s^#1L-2AL recombinant line	SFL0.70-LFL0.87	Liu et al. (2017) [7]
89-152 ^4^	CS-*Ae. searsii* TiW?S-2S^s^#1S-W?S.W?L recombinant line	SFL0.35-0.82	Liu et al. (2017) [7]
89-185	CS-*Ae. searsii* ditelosomic 2S^s^#1L addition line	LFLC-1.00	Liu et al. (2017) [7]
89-346	CS-*Ae. searsii* T2BS.2BL-2S^s^#1L recombinant line	LFL0.72-1.00	Liu et al. (2017) [7]
89-378 ^4^	CS-*Ae. searsii* T2S^s^#1S.2S^s^#1L-2W? recombinant line	SFL1.00-LFL0.64	Liu et al. (2017) [7]

^1^ WGRC: Wheat Genetics Resource Center at Kansas State University, Manhattan, KS, USA; ^2^ with no 2S^s^#1 segment; ^3^ unknown authorship; ^4^ the wheat chromosome involved in the recombination was not assigned (represented by W?). LFL = long-arm fraction length; SFL = short-arm fraction length.

**Table 2:** Molecular markers used to identify recombinants and physically map *Pm57*.

Locus ^1^ No.	Marker Name	Chromosome Bin	Genomic Position (Mb) ^2^
1	*X23241*	LFLC-0.64	488.87
2	*X216815*	LFC0.64-0.67	658.61
3	*X170551*	LFL0.72-0.87	320.50
4	*X123945*	LFL0.72-0.87	622.52
5	*X185442*	LFL0.72-0.87	733.47
6	*X305960*	LFL0.72-0.87	744.52
7	*X26866*	LFL0.72-0.87	752.95
8	*X284274*	LFL0.72-0.87	748.97
9	*X37693*	LFL0.72-0.87	754.92
10	*X251565*	LFL0.72-0.87	777.64
11	*X103528*	LFL0.72-0.87	762.50
12	*X5286/X31536* ^3^	LFL0.72-0.87	765.05
13	*X33739*	LFL0.72-0.87	766.23
14	*X268081*	LFL0.72-0.87	768.61
15	*X49873*	LFL0.72-0.87	770.18
16	*X256410*	LFL0.72-0.87	771.04
17	*X67593*	LFL0.72-0.87	773.72
18	*X33705*	LFL0.72-0.87	775.02
19	*X430422/X285619/X205834/X240724* ^3^	LFL0.72-0.87	775.33
20	*X4364*	LFL0.72-0.87	778.33
21	*X62492*	LFL0.72-0.87	778.85
22	*X55871*	LFL0.72-0.87	779.34
23	*X23212*	LFL0.87-1.00	783.60
24	*X2L4g9p4*	LFL0.87-1.00	788.66

^1^ Unigenes from which makers are derived; ^2^ corresponds to the location on the wheat chromosome 2B in CS RefSeq v1.1 (IWGSC, 2018); ^3^ markers designed based on the sequence of the same gene.

## Supplementary Materials

Supplementary materials can be found at https://www.mdpi.com/1422-0067/21/1/322/s1. Table S1 List of unigenes of specifically expressed in 2011-400; Table S2 List of unigenes shared by all three *Pm57* carrying lines; Table S3 List of genes and primers for qRT-PCR; Table S4 Results of qRT-PCR; Table S5 List of molecular markers of *Ae. searsii* chromosome 2S^s^#1 specificity and derived transcriptome unigenes used in this study; Table S6 List of CS-*Ae. searsii* 2S^s^#1 recombinants newly identified, their molecular markers presenting, and response to *Bgt*-infection; Table S7 Collineary analysis of *Pm57* mapped genomic region with related species.

## Abbreviations

*Bgt*	*Blumeria graminis* f. sp. *tritici*
FL	fraction length
CNL	coiled-coil nucleotide-binding site-leucine-rich repeat
DEG	differential expression gene
SFL	short-arm fraction length
LFL	long-arm fraction length
RPKM	reads per kilobase per million mapped reads
IT	infection types
VIGS	virus-induced gene silencing
